# Mental and Physical Health-Related Risk Factors Among Females Who Died by Firearm Suicide

**DOI:** 10.1001/jamanetworkopen.2025.5941

**Published:** 2025-04-18

**Authors:** Laura C. Prater, Pejmon Noghrehchi, Ning Duan, Julian Takagi-Stewart, Stephen J. Mooney, Jennifer L. Hefner, Evan V. Goldstein

**Affiliations:** 1 Division of Health Services Management and Policy, College of Public Health, The Ohio State University, Columbus; 2 Department of Psychiatry and Behavioral Health, University of Washington, Seattle; 3 Firearm Injury & Policy Research Program, Harborview Medical Center, University of Washington School of Medicine, Seattle; 4 Department of Mathematics and Statistics, University of Massachusetts Amherst; 5 Drexel University College of Medicine, Philadelphia, Pennsylvania; 6 Department of Epidemiology, School of Public Health, University of Washington, Seattle; 7 Department of Population Health Sciences, University of Utah School of Medicine, Salt Lake City

## Abstract

**Importance:**

Firearm suicide among females has increased in the past decade; routes for prevention remain unclear.

**Objective:**

To identify classes of firearm suicide decedents among females based on preceding mental and physical health-related risk factors for suicide.

**Design, Setting, and Participants:**

This cross-sectional retrospective study of females who died by firearm suicide between January 2014 and December 2018 used state-based surveillance data from the National Violent Death Reporting System Restricted Access Database representing 1 or more years of data from all 50 US states, the District of Columbia, and Puerto Rico; data were analyzed from March 2022 to September 2023. A latent class analysis was used to identify and characterize categories of mental and physical health-related factors among females (ie, classes).

**Exposure:**

Known mental and physical health-related risk factors for suicide.

**Main Outcome and Measure:**

The numbers (percentages) of females who died by firearm suicide across various factors.

**Results:**

Of the 8318 female decedents in our sample (mean [SD] age, 47.2 [17.0] years), more than half (4816 of 8318 [57.9%]) had more than 1 risk factor endorsed. Among this subgroup, a 4-class model was identified: (1) alcohol use disorder or substance use disorder (1273 [26.4%]); (2) depression and suicidal thoughts (2289 [47.5%]); (3) physical health problems and pain (1054 [21.9%]); and (4) all conditions (multimorbid; 200 [4.2%]); 3502 (42.1%) could not be classified. Black females made up a higher proportion (244 of 3502 [7.0%]) of the females with a 0 or 1 risk factor endorsed than of the females with multiple risk factors (184 of 4816 [3.8%]). Mental health problems were apparent in 51.7% of the full sample (4303 of 8318), with 28.6% (n = 2376) demonstrating evidence of mental health treatment. More than one-fifth (1766 of 8318 [21.2%]) had physical health problems. More than one-fourth of the full sample (2239 of 8318 [26.9%]) and nearly 40% (505 of 1273 [39.7%]) in class 1 had intimate partner problems before their death.

**Conclusions and Relevance:**

In this cross-sectional study of females who died by firearm suicide, meaningfully distinct groups of female firearm suicide decedents were identified. Given the high proportion of females without mental health and physical health problems, it is plausible that many female firearm suicide decedents did not frequently intersect with the health care system before their death. Results suggest opportunities for preventing firearm suicide among females through nonmedical care settings.

## Key Points

**Question:**

Are there distinct profiles of mental and/or physical illness and suicidal behaviors (ie, thoughts, intent, and attempts) among females who died by firearm suicide?

**Findings:**

In this cross-sectional study, 57.9% of cases of firearm suicides among females were represented in 1 of 4 profiles: alcohol or substance use disorders, depression and suicidal thoughts, physical health problems and pain, and multimorbidity; 42.1% of cases could not be classified.

**Meaning:**

These findings suggest that in the cases that were not classified, the females had relatively low proportions of documented mental or physical health problems, which may indicate low engagement with health care settings and may require intervention in other community settings.

## Teaser

This cross-sectional study identifies classes of firearm suicide decedents among females based on preceding mental and physical health-related risk factors for suicide between 2014 and 2018.

## Introduction

Suicide is a critical health problem in the US and a leading cause of death for females through middle age.^1^ Roughly one-third of all suicides among females are caused by a firearm, a proportion that has increased by approximately 28% in the past 2 decades.^1,2,3^ This increase can be associated with the statistic that first-time firearm ownership has increased in recent years, and approximately half of the new owners have been females.^4^ Additionally, females who die by firearm suicide are more likely than men to use a firearm belonging to another person in the home, particularly that of an intimate partner.^5^ As with other populations,^4,6,7,8^ preventing suicide death among females continues to be a challenge—and one that is further complicated by the evolving dynamics of firearm access by females.

Often identified in health care encounters, mental and physical health problems, substance use disorders, and a history of suicidal behavior are common risk factors for suicide.^9,10^ Understanding common patterns of co-occurring health-related risk factors among females who died by firearm suicide may allow for specific health care settings and types of health care professionals to be prioritized for intervention development. A focus on females is necessary given the unique characteristics of firearm ownership among females, including the high proportion of females who die by firearm suicide with a firearm under the control of someone else in the home.^5^

The first step toward effective female-centered interventions for firearm suicide prevention is to identify for whom, and at what level, the problem exists. This work is part of step 0 of the National Institutes of Health Stage Model for Behavioral Intervention Development,^11^ in which basic science translatable to intervention development is used to explore the nature and extent of the problem.^12^ The ideal scenario is to identify specific clinical typologies of female firearm suicide decedents to develop effective targeted interventions. Our study addresses this goal by examining discernable patterns of clinically relevant risk factors for female firearm suicide decedents to identify categories of females (classes) as potential targets for intervention within the health care system.

## Methods

### Data Source

Through a restricted access request,^13^ we used data from the Centers for Disease Control and Prevention National Violent Death Reporting System (NVDRS) Restricted Access Database^14^ to examine all-age firearm suicides among females from January 2014 to December 2018. State participation in the NVDRS increased over the study period; all 50 US states, Puerto Rico, and Washington, DC, were included in our study for at least 1 study year. States in our sample and years of data reported are provided in the eTable in Supplement 1. The NVDRS contains data from multiple sources, including death certificates, reports from coroners or medical examiners, and law enforcement reports, including next-of-kin interviews, clinical data, and court records. For each decedent, the NVDRS generally reports both abstracted and coded variables, as well as narrative summaries of coroners or medical examiners and law enforcement investigation reports, although, in some cases, 1 or both narrative reports are missing. We used preexisting coded variables from the NVDRS^15^ and new variables coded via natural language processing of the incident narratives (eg, acute or chronic pain or romantic relationship problem)—methods described in detail in earlier work.^16^ Guidance from University of Washington’s institutional review board indicated that this study did not meet requirements for human research, specifically the use of administrative data from deceased individuals; therefore, informed consent was not sought. Result reporting adhered to the Strengthening the Reporting of Observational Studies in Epidemiology (STROBE) reporting guideline^17^ for cross-sectional designs.

### Measures

We used a clinically guided approach informed by risk factors for suicide when selecting variables from the NVDRS to identify our latent construct of clinical typologies among females. Our process focused on observable variables that identified mental and physical health diagnoses, treatment seeking, and suicidal behaviors commonly addressed in health care settings. The following risk factors were used to generate our classes: anxiety, bipolar disorder, depression, posttraumatic stress disorder, schizophrenia, alcohol use disorder, substance use disorder, suicidal thoughts, stated suicidal intent, suicide attempt, physical health problem, and acute or chronic pain. The Centers for Disease Control and Prevention NVDRS abstractors coded a set list of mental health conditions per the *Diagnostic and Statistical Manual of Mental Disorders* (Fifth Edition)^18^ as noted in the investigation reports. Physical health problems and pain were coded if present in the investigation reports and if a contributing factor to the suicide. Mental illness and alcohol use disorder or substance use disorder were coded if abstractors observed them in the investigation reports, regardless of whether it contributed to the death. Finally, suicidal thoughts or intent and behaviors (ie, attempts) were coded if mentioned in the investigation reports, usually from next-of-kin interviews or suicide notes.

To further describe our classes, we assessed demographic characteristics (eg, age, education, and race and ethnicity) and other circumstantial variables (eg, financial problems and intimate partner problems) not directly informing the latent construct. Demographic characteristics were endorsed from the death certificate and, in some cases, investigation reports. Abstractors coded these variables if it was clear from the investigation reports that they were related to or immediately preceded the death or were impending. The NVDRS compiles race and ethnicity data from multiple primary source documents, specifically from death certificates, coroner or medical examiner reports, and law enforcement reports. Next-of-kin informants generally report the decedent’s race and ethnicity but are sometimes based on observations by coroners, medical examiners, or funeral directors when informants are unavailable.^19^ Race categories included American Indian or Alaska Native; Asian, Native Hawaiian, or Other Pacific Islander; Black or African American; 2 or more races; White; unknown; or unspecified (decedent’s ethnicity was provided in place of their race, and no other value was given). NVDRS abstractors code decedents as 2 or more races when source documents indicate multiple races, which aligns with the standards used by the US Census Bureau.^15^ Ethnicity is a separate field in the NVDRS database and is endorsed independently of race. Ethnicity is categorized as Hispanic or Latino, non-Hispanic or non-Latino, or unknown. Race and ethnicity were examined to identify potential disparities in the availability and endorsement of circumstantial variables.

### Statistical Analysis

We conducted a latent class analysis (LCA) to generate clinical typologies (ie, latent classes) of female firearm suicide decedents using observable response variables. The manifest variables used to construct these classes included mental illness, physical health problems, and suicidal thoughts and behaviors.^20^ LCA is a popular modeling approach in health services research for its ability to identify population subgroups that represent clinically meaningful typologies.^21,22^ LCA produces estimates for each individual of the probability of belonging to a specific class and class-specific item endorsement probabilities for each measure.^23^ To select the appropriate number of classes, we compared 2- to 6-class models and assessed the Akaike information criterion, bayesian information criterion, and entropy to create a parsimonious and interpretable model to inform prevention strategies. We specified 200 draws and allowed 20 iterations for each model. The “gsem” command in StataBE, version 17.0 (StataCorp LLC) was used to perform the LCA,^24^ and the statistical analysis was performed from September 2021 to August 2022.

We ran a separate LCA for each study year (2014-2018) and compared more recent years (2017 and 2018) with earlier years (2014-2016) to understand the temporal stability of our final model in terms of NVDRS data procedures as well as secular trends. We also ran the model across 3 random seeds to ensure our estimates were consistent. To aid in a final decision between class numbers performing similarly, we assessed subject movement from parsimonious models to higher-order class numbers. We compared class membership over time as a rough measure of class allocation over each year in the study. We report proportions and means of relevant demographic and circumstantial characteristics across the 4 classes.

## Results

There were 8318 female firearm suicide decedents (mean [SD] age, 47.2 [17.0] years; 428 [5.2%] Black or African American, 302 [3.6%] Hispanic or Latino; and 7534 [90.6%] White) included in the NVDRS from 2014 to 2018. Our LCA initially resulted in classes that were not interpretable, demonstrating relatively poor fit statistics using the full sample due to the high proportion (n = 3502 [42.1%]) of females who endorsed either 0 or 1 of the class characteristics. We therefore omitted these 3502 females and described them separately alongside females with multiple endorsements, as shown in Table 1. Females with 0 or 1 characteristic endorsed were demographically similar to those with multiple characteristics endorsed apart from race—Black females were overrepresented (244 of 3502 [7.0%]) relative to the proportion in the full sample (428 of 8318 [5.1%]). Mental health diagnoses and treatment, substance use disorders, and most circumstantial variables were less commonly reported among females with 0 or 1 endorsed characteristic relative to females with multiple endorsed characteristics. Intimate partner problems were the exception, with a relatively high proportion of both excluded (23.4% [818 of 3502]) and included (29.5% [1421 of 4816]) females experiencing them before suicide.

**Table 1.** Characteristics of 8318 Female Suicide Decedents, 2014-2018

Characteristic	Females, No. (%)		
	Overall (N = 8318 [100%])	0 or 1 Risk factor (n = 3502 [42.1%])	Multiple risk factors (n = 4816 [57.9%])
Mean (SD) age, y^a^	47.19 (17.0)	46.65 (17.5)	47.59 (16.5)
Race and ethnicity			
American Indian or Alaska Native	96 (1.2)	41 (1.2)	55 (1.1)
Asian or Other Pacific Islander	125 (1.5)	63 (1.8)	62 (1.3)
Black or African American	428 (5.2)	244 (7.0)	184 (3.8)
Hispanic or Latino	302 (3.6)	120 (3.4)	182 (3.8)
≥2 Races	71 (0.9)	27 (0.8)	44 (0.9)
White	7534 (90.6)	3097 (88.4)	4437 (92.1)
Unknown^b^	11 (0.1)	10 (0.3)	1 (0.0)
Unspecified^c^	53 (0.6)	20 (0.6)	33 (0.7)
Marital status			
Single, divorced, separated	5067 (60.9)	2183 (62.3)	2884 (59.9)
Married, civil union, domestic partnership	3251 (39.1)	1319 (37.7)	1932 (40.1)
Ever served in military			
Yes	338 (4.1)	131 (3.7)	207 (4.3)
No	7980 (95.9)	3371 (96.3)	4609 (95.7)
Education level			
Less than high school	885 (10.6)	417 (11.9)	468 (9.7)
High school or greater	7433 (89.4)	3085 (88.1)	4348 (90.3)
Mental or physical health			
Alcohol use disorder	1792 (21.5)	121 (3.5)	1671 (34.7)
Substance use disorder^d^	1034 (12.4)	S	1034 (21.5)
Current mental health problem	4303 (51.7)	807 (23.0)	3496 (72.7)
Diagnosis^d^^,^^e^			
Depression	3363 (40.4)	483 (13.8)	2880 (59.8)
Anxiety	939 (11.3)	38 (1.1)	901 (18.7)
Bipolar	680 (8.2)	43 (1.2)	637 (13.2)
Eating disorder	23 (0.3)	S	17 (0.4)
Obsessive compulsive disorder	20 (0.2)	S	17 (0.4)
Posttraumatic stress disorder	143 (1.7)	11 (0.3)	132 (2.7)
Schizophrenia	119 (1.4)	16 (0.5)	103 (2.1)
Unknown^f^	343 (4.1)	174 (5.0)	169 (3.5)
Other^g^	230 (2.8)	59 (1.7)	171 (3.6)
Undergoing mental health treatment	2376 (28.6)	392 (11.2)	1984 (41.2)
Current physical health problem^d^	1766 (21.2)	275 (7.9)	1491 (31.0)
Suicidal intent^d^	1844 (22.2)	185 (5.3)	1659 (34.5)
History of suicidal thoughts^d^	2676 (32.2)	164 (4.7)	2512 (52.2)
History of suicide attempts^d^	1827 (22.0)	152 (4.3)	1675 (34.8)
Circumstances			
Intimate partner problem	2239 (26.9)	818 (23.4)	1421 (29.5)
Recent suicide of friend or family	255 (3.1)	65 (1.9)	190 (4.0)
Other death of friend or family	622 (7.5)	234 (6.7)	388 (8.1)
Job problem	578 (7.0)	205 (5.9)	373 (7.8)
Financial problem	632 (7.6)	216 (6.1)	416 (8.6)
Eviction or loss of home	277 (3.3)	96 (2.7)	181 (3.8)
Traumatic anniversary	73 (0.9)	30 (0.9)	43 (0.9)
Exposure to disaster	S	S	S
Recent criminal or legal problem	248 (3.0)	94 (2.7)	154 (3.2)
Acute or chronic pain^d^	1207 (14.5)	75 (2.1)	1132 (23.5)
Recent dispute	1618 (19.5)	561 (16.0)	1057 (22.0)
Romantic relationship problem	1563 (18.8)	552 (15.8)	1011 (21.0)

Abbreviation: S, suppressed because of cell count of 10 or less.

^a^ Age missing for 1 decedent.

^b^ Indicates that it is unknown whether individual decedent was or was not of Hispanic or Latino origin.^15^

^c^ Indicates that individual decedent’s ethnicity was provided in place of their race and no other value was given.^15^

^d^ Indicates variables used to construct the latent class analysis.

^e^ Diagnosis is only indicated if “yes” to “current mental health problem” (n = 4303).

^f^ Unknown indicates that the individual had a mental health diagnosis but that the diagnosis was not listed.

^g^ ”Other” indicates that the diagnosis was not on the code list used by the National Violent Death Reporting System.

By balancing bayesian information criterion and entropy (Table 2), the 4-class model was selected using the group of females (n = 4816 [57.9%]) with multiple characteristics endorsed. The classes, as shown in Table 3, included class 1, defined by females with a substance use or alcohol disorder; class 2, defined by females with a history of depression and suicidal thoughts; class 3, defined by females experiencing pain or a physical health problem; and class 4, defined by females with a high probability of all variables used to create our classes (ie, multimorbid). In the sample used for our LCA (n = 4816), class 2 was the largest with 2289 females (47.5%), followed by class 1 (1273 [26.4%]), class 3 (1054 [21.9%]), and class 4, which was the smallest (200 [4.2%]).

**Table 2.** Model Fit Statistics

Model	Akaike information criterion	Bayesian information criterion	Entropy
2 Class	55550.71	55706.23	0.98354524
3 Class	54147.52	54387.26	0.94441907
4 Class	53884.59	54208.57	0.93894854
5 Class	53323.45	53731.67	0.90207056
6 Class	53040.61	53539.55	0.89574135

**Table 3.** Probabilities of Mental and Physical Health Risk Factors Among 4816 Female Individuals Who Died From Suicide in 2014-2018 in the National Violent Death Reporting System^a^

Risk factor^b^	LCA proportions (weighted)			
	Class 1: alcohol or substance use disorder (n = 1273 [26.4%])	Class 2: depression or suicidal thoughts (n = 2289 [47.5%])	Class 3: physical health problems or pain (n = 1054 [21.9%])	Class 4: all conditions (n = 200 [4.2%])
Anxiety	0.12	0.24	0.14	0.26
Bipolar	0.12	0.19	0.05	0.10
Depression	0.49	0.73	0.44	0.59
Posttraumatic stress disorder	0.03	0.04	0.02	0.03
Schizophrenia	0.02	0.04	NA	0.03
Alcohol use disorder	1.00	0.09	0.06	1.00
Substance abuse disorder	0.71	0.01	0.01	0.86
History of suicidal thoughts	0.46	0.61	0.37	0.65
Suicidal intent	0.31	0.41	0.24	0.40
History of suicidal attempts	0.31	0.44	0.18	0.40
Physical health problem	0.05	0.12	0.96	0.81
Acute chronic pain	0.07	0.10	0.65	0.70

Abbreviations: LCA, latent class analysis; NA, not applicable.

^a^ Marginal probabilities reflect class-specific endorsement probabilities for variables used to generate classes.

^b^ These risk factors were used to generate the classes.

Table 4 displays demographic and circumstantial characteristics across the 4 classes. Class 1 had the highest proportion of unmarried females relative to classes 2, 3, and 4 (68.3% [870 of 1273] vs 60.4% [1382 of 2289], 50.8% [535 of 1054], and 48.5% [97 of 200], respectively) and the highest proportion of intimate partner problems (39.7% [505 of 1273] vs 31.8% [727 of 2289], 11.6% [122 of 1054], and 33.5% [67 of 200], respectively) and recent disputes (35.6% [453 of 1273] vs 20.3 [465 of 2289], 8.8% [93 of 1054], and 23.0% [46 of 200], respectively). Females in class 2 had the highest proportion of Black women at 5.3% (122 of 2289), which was 2 to 3 percentage points higher than the other classes. Relative to classes 1, 2, and 4, class 3 females were older (mean [SD] age, 59.4 [15.0] years vs 41.9 [13.5], 45.2 [16.4], and 49.8 [13.4] years, respectively) and had lower proportions of circumstantial events such as intimate partner problems (11.6% [122 of 1054] vs 39.7% [505 of 1273], 31.8% [727 of 2289], and 33.5% [67 of 200], respectively) and recent disputes (8.8% [93 of 1054] vs 35.6% [453 of 1273], 20.3% [465 of 2289], and 23.0% [46 of 200], respectively). Relative to classes 1, 2 and 3, females in class 4 had high proportions of financial problems (16.0% [32 of 200] vs 9.1% [116 of 1273], 8.2% [188 of 2289], and 7.6% [80 of 1054], respectively) and job problems (11.5% [23 of 200] vs 9.1% [116 of 1273], 8.2% [187 of 2289], and 4.5% [47 of 1054], respectively). In terms of known use of mental health services, approximately half (50.7% [1161 of 2289) of females in class 2 and about one-third (33.5% [426 of 1273]) of females in class 1 were noted as having received mental health services prior to death. Table 5 shows the allocation of the 4816 females assigned to a class within our study. Class allocation remained relatively steady, with the largest changes in relative size of class membership occurring in class 1 between 2014 and 2015, a decrease of 7.5 percentage points (n = 22). Differences in 2016 and beyond stayed at 3 percentage points or lower, apart from movement in class 3 between 2015 and 2016 (n = 74 [3.2 percentage points]).

**Table 4.** Demographic and Circumstantial Characteristics Across 4-Class Latent Class Model

Variable	Females, No. (%)			
	Class 1: alcohol or substance use disorder (n = 1273 [26.4%])	Class 2: depression or suicidal thoughts (n = 2289 [47.5%])	Class 3: physical health problem or pain (n = 1054 [21.9%])	Class 4: all conditions (n = 200 [4.2%])
Mean (SD) age, y	41.9 (13.5)	45.2 (16.4)	59.4 (15.0)	49.8 (13.4)
Race				
American Indian or Alaska Native	20 (1.6)	23 (1.0)	S	S
Asian or Other Pacific Islander	11 (0.9)	40 (2.0)	S	S
Black or African American	33 (2.6)	122 (5.3)	25 (2.4)	S
Unspecified^a^	S	17 (0.7)	S	S
≥2 Races	15 (1.2)	20 (0.9)	S	S
White	1189 (93.4)	2066 (90.3)	997 (94.6)	185 (92.5)
Ethnicity				
Hispanic or Latino	55 (4.3)	96 (4.2)	25 (2.4)	S
Non-Hispanic or non-Latino	1218 (95.7)	2193 (95.8)	1026 (97.6)	194 (97.0)
Unknown^b^	S	S	S	S
Marital status				
Single	870 (68.3)	1382 (60.4)	535 (50.8)	97 (48.5)
Married, civil union, domestic partnership	403 (31.7)	907 (39.6)	519 (49.2)	103 (51.5)
Ever served in military				
Yes	52 (4.1)	103 (4.5)	40 (3.8)	12 (6.0)
No	1221 (95.9)	2186 (95.5)	1014 (96.2)	188 (94.0)
Education level				
Less than high school	12 (0.9)	237 (10.4)	70 (6.6)	19 (9.5)
High school or greater	1261 (99.1)	2052 (89.6)	984 (93.4)	181 (90.5)
Circumstantial variable				
Undergoing mental health treatment	426 (33.5)	1161 (50.7)	298 (28.3)	99 (49.5)
Intimate partner problem	505 (39.7)	727 (31.8)	122 (11.6)	67 (33.5)
Recent suicide of friend or family	67 (5.3)	78 (3.4)	33 (3.1)	12 (6.0)
Other death of friend or family	115 (9.0)	175 (7.7)	79 (7.5)	19 (9.5)
Job problem	116 (9.1)	187 (8.2)	47 (4.5)	23 (11.5)
Financial problem	116 (9.1)	188 (8.2)	80 (7.6)	32 (16.0)
Eviction or loss of home	69 (5.4)	71 (3.1)	32 (3.0)	S
Traumatic anniversary	13 (1.0)	19 (0.8)	S	S
Exposure to disaster	S	S	S	S
Recent criminal or legal problem	84 (6.6)	50 (2.2)	15 (1.4)	S
Recent dispute	453 (35.6)	465 (20.3)	93 (8.8)	46 (23.0)

Abbreviation: S, suppressed because of cell count of 10 or less.

^a^ Indicates that individual decedent’s ethnicity was provided in place of their race and that no other value was given.^15^

^b^ Indicates that it is unknown whether individual decedent was or was not of Hispanic or Latino origin.^15^

**Table 5.** Class Allocation by Year Among 4816 Female Suicide Decedents in the National Violent Death Reporting System Between 2014 and 2018

Class of risk factor patterns	Females, No. (%)				
	2014 (n = 561)	2015 (n = 805)	2016 (n = 1008)	2017 (n = 1142)	2018 (n = 1300)
1: Alcohol or substance use disorder	191 (34.0)	213 (26.5)	258 (25.6)	281 (24.6)	330 (25.4)
2: Depression or suicidal thoughts	241 (43.0)	397 (49.3)	470 (46.6)	542 (47.5)	639 (49.2)
3: Physical health problem or pain	113 (20.1)	166 (20.6)	240 (23.8)	256 (22.4)	279 (21.5)
4: All conditions (multimorbid)	16 (2.9)	29 (3.6)	40 (4.0)	63 (5.5)	52 (4.0)

## Discussion

Although previous studies^25,26^ have demonstrated that individual risk factors—such as depression, substance abuse, and intimate partner violence (IPV)—are essential for understanding suicide among females, detecting risks and preventing suicide death present ongoing challenges for many clinicians, public health professionals, and families. Suicide is a multifactorial, polygenic phenotype with simultaneous biopsychological, social, and environmental contributors, and firearm availability exacerbates suicide risk for persons with suicidal ideation.^27,28,29,30^ Increasing rates of firearm suicide death among females and changing patterns in the breadth of firearm access among females underscore the need for a better understanding of the profile of females at risk, including an assessment of the circumstantial and demographic traits common among these profiles. In addressing this challenge, we identified 4 classes of female firearm suicide decedents, each characterized by a distinct combination of health characteristics: (1) alcohol use disorder or substance use disorder (26.4%); (2) depression and suicidal thoughts (47.5%); (3) physical health problems and pain (21.9%); and (4) all risk factors or multimorbid (4.2%). The following paragraphs consider the implications of these 4 classes for intervention development and targeting.

Our findings reiterate that suicide deaths are not exclusively driven by mental illness or substance use disorder. In fact, physical health and social factors often precipitate suicide attempts.^6,31^ A burgeoning evidence basis demonstrates that mental health diagnoses, such as depression, may be less likely among persons who die by suicide using firearms relative to other means.^4,8,9^ Our LCA underscored this nuance and complexity, which may imply that we need to focus more on lethal means interventions and less on mental health counseling for suicide prevention. Previous studies^2,10,16,32^ suggest lethal means assessment and counseling may improve safe firearm storage, reduce firearm suicide attempts, and improve opportunities to treat underlying health needs. Although many clinicians do not routinely screen or counsel patients on firearm safety,^25^ training for lethal means assessment and counseling can improve health care professionals’ willingness to discuss firearms and conduct lethal means assessment with their patients.^28,33^

Still, depression, depressive symptoms, and substance abuse are often important risk factors for suicidality.^6^ Depression with a history of suicidal thoughts and intent were the definitive characteristics of class 2 decedents, the largest class, representing 47.5% of the sample used in our LCA. However, consistent with prior evidence demonstrating that mental illness often goes undiagnosed before suicide death,^29^ only about half (50.7%) of the class 2 decedents were known to interact with mental health care services before death. Even fewer decedents (33.5%) in class 1—characterized by their substance or alcohol use disorder—were undergoing mental health treatment before death. Of relevance to these findings, extended literature^34,35^ demonstrates the difficulty persons experiencing mental illness and substance use have in accessing care or adhering to medical treatment. Reaching these individuals in clinical settings will likely be an ongoing challenge.

Local health care systems, however, can commit to strategically addressing this challenge by implementing evidence-based suicide prevention models such as Zero Suicide,^36^ a system-wide framework committed to systematically improving training and lethal means assessment. Additionally, females resembling those represented in classes 1 and 2 may benefit from community-based approaches to lethal means assessment and counseling, including intervention by nonprofit human service agencies, permanent supportive housing and homeless service agencies, or other settings where women seek help for substance use disorders. Given the high percentage of class 1 decedents who experienced problems with intimate partners, brief lethal means assessment and counseling may be particularly effective if delivered by organizations providing free and confidential services to females who have experienced IPV—a specific manifestation of intimate partner problems—as well as through the National Domestic Violence Hotline.

Class 3 represented 1 in 5 (21.9%) of decedents included in our analyses who were characterized by their experiences of pain or physical health problems. Class 3 may, therefore, indicate an opportunity to broaden the criteria for initiating lethal means assessment interventions with female patients who experience not only mental illness or suicidal ideation but also chronic physical conditions. Given the operational challenges associated with expanding screening services into current clinical practice, it may be unrealistic for many health care professionals to expand lethal means assessment to such a broad category of females. Despite these obstacles, our findings lend support for integrating targeted interventions in nonpsychiatric care settings using nonpsychiatric care professionals who significantly interact with this profile of female patients at risk.^37^ More specifically, physical and occupational therapists (PTs and OTs) are positioned well to identify suicidal ideation given their more frequent interactions with patient populations experiencing pain or other physical health problems compared with mental health professionals. As such, PTs, OTs, and other rehabilitative specialists and settings not only serve as a crucial link between patients and mental health services but show promise in their ability to provide targeted suicide prevention interventions for class 3 female patients.^38,39^

Class 4 emphasized the complicated and polygenic nature of suicidality, as well as a reality many clinicians face: for some patients, detecting suicidality and preventing suicide death can feel like impossible undertakings, especially when highly lethal firearm attempts are involved. Clinician time constraints and competing clinical responsibilities are critical barriers to expanding lethal means assessment to more patients.^40,41^ Additional research is needed to investigate the subpopulation of females experiencing multimorbid mental and physical health conditions, including both decedents and living persons, compared with controls. Novel machine learning techniques coupled with clinician assessment may help improve the detection of suicide risk among these women with greater precision and sensitivity.^42,43^ In the meantime, females resembling decedents represented in class 4 may benefit from the spillover effects of a strengthened commitment to expanding lethal means assessment and counseling to females experiencing depression, substance use disorder, and/or chronic physical health conditions and pain (ie, classes 1, 2, and 3) in health care and community settings.

### Limitations

There were several limitations to our study. The NVDRS is a national collection of state-based surveillance program data of violent deaths formed by the linkage of coroner or medical examiner, law enforcement, and toxicology reports. These data rely on what is reported in death records provided to the Centers for Disease Control and Prevention and often represent information provided by next-of-kin interviews, subject to recall bias and potential misclassification (particularly the underreporting of circumstantial variables). IPV is reported only for states using the optional IPV module. Intimate partner problems—the variable used in our study—is broader, indicating arguments and divorce, among other problems. Although all 50 US states, the District of Columbia, and Puerto Rico reported at least 1 year of data during our study period, participation generally increased over time. Not all states or territories reported all 5 years of data, limiting generalizability. Focusing on the 5 most recent years of data at the time of study initiation was an attempt to minimize the effect of differential reporting years by states. Additionally, Black women comprised 5.2% of the general sample but were overrepresented among those with 0 or 1 characteristic (7.0%) available, as noted in Table 1. Prior literature^44^ has demonstrated that Black individuals are overrepresented among those with short coroner or medical examiner and law enforcement reports and among those missing law enforcement reports altogether. Our study may support and reinforce that less information is generally available for Black female firearm suicide decedents relative to White women, but more research is needed to confirm this observation.

## Conclusions

In this cross-sectional retrospective study of women who died by firearm suicide between 2014 and 2018, 4 groups of female firearm suicide decedents were uncovered, among whom mental and physical health problems were common. For these women, the integration of lethal means assessment—particularly firearm safety assessments—was important in varied health care venues. A large proportion of our sample had limited health-related data available, indicating a disparity in information across female firearm suicide decedents that must be addressed through additional research. Intimate partner problems were common across our sample, indicating the need to coordinate firearm safety and IPV prevention in tandem and across intervention settings.

## Notes

Supplement 1. **eTable.** States and Years Reporting Into Study Period, 2014-2018

Supplement 2.
Data Sharing Statement

## References

[1] Centers for Disease Control and Prevention. Web-based Injury Statistics Query and Reporting System (WISQARS). Accessed February 1, 2024. https://wisqars.cdc.gov/

[2] Anestis MD. Prior suicide attempts are less common in suicide decedents who died by firearms relative to those who died by other means. J Affect Disord. 2016;189:106-109. doi:10.1016/j.jad.2015.09.007 26432034

[3] Shenassa ED, Catlin SN, Buka SL. Lethality of firearms relative to other suicide methods: a population based study. J Epidemiol Community Health. 2003;57(2):120-124. doi:10.1136/jech.57.2.120 12540687 PMC1732374

[4] Bahraini N, Brenner LA, Barry C, . Assessment of rates of suicide risk screening and prevalence of positive screening results among us veterans after implementation of the veterans affairs suicide risk identification strategy. JAMA Netw Open. 2020;3(10):e2022531. doi:10.1001/jamanetworkopen.2020.22531 33084900 PMC7578771

[5] Barber C, Azrael D, Miller M, Hemenway D. Who owned the gun in firearm suicides of men, women, and youth in five US states? Prev Med. 2022;164:107066. doi:10.1016/j.ypmed.2022.107066 35461957

[6] Kline-Simon AH, Sterling S, Young-Wolff K, . Estimates of workload associated with suicide risk alerts after implementation of risk-prediction model. JAMA Netw Open. 2020;3(10):e2021189. doi:10.1001/jamanetworkopen.2020.21189 33084896 PMC7578770

[7] Perlis RH, Fihn SD. Hard truths about suicide prevention. JAMA Netw Open. 2020;3(10):e2022713. doi:10.1001/jamanetworkopen.2020.22713 33084895

[8] Wyman PA, Pisani AR, Brown CH, . Effect of the wingman-connect upstream suicide prevention program for air force personnel in training: a cluster randomized clinical trial. JAMA Netw Open. 2020;3(10):e2022532. doi:10.1001/jamanetworkopen.2020.22532 33084901 PMC7578767

[9] Liu Q, Wang X, Kong X, . Subsequent risk of suicide among 9,300,812 cancer survivors in US: a population-based cohort study covering 40 years of data. EClinicalMedicine. 2022;44:101295. doi:10.1016/j.eclinm.2022.101295 35198920 PMC8850339

[10] Swanson JW, McGinty EE, Fazel S, Mays VM. Mental illness and reduction of gun violence and suicide: bringing epidemiologic research to policy. Ann Epidemiol. 2015;25(5):366-376. doi:10.1016/j.annepidem.2014.03.004 24861430 PMC4211925

[11] National Institute on Aging. NIH Stage Model for Behavioral Intervention Development. Updated January 11, 2024. Accessed February 1, 2024. https://www.nia.nih.gov/research/dbsr/nih-stage-model-behavioral-intervention-development

[12] Onken LS, Carroll KM, Shoham V, Cuthbert BN, Riddle M. Reenvisioning clinical science: unifying the discipline to improve the public health. Clin Psychol Sci. 2014;2(1):22-34. doi:10.1177/2167702613497932 25821658 PMC4374633

[13] Centers for Disease Control and Prevention. NVDRS data access. Centers for Disease Control and Prevention, National Centers for Injury Prevention and Control. Updated May 16, 2024. Accessed October 1, 2021. https://www.cdc.gov/nvdrs/about/nvdrs-data-access.html?CDC_AAref_Val=https://www.cdc.gov/violenceprevention/datasources/nvdrs/dataaccess.html

[14] Centers for Disease Control and Prevention. National Violent Death Reporting System (NVDRS). Centers for Disease Control and Prevention. Updated February 1, 2022. Accessed February 1, 2024. https://www.cdc.gov/nvdrs/about/?CDC_AAref_Val=https://www.cdc.gov/violenceprevention/datasources/nvdrs/index.html

[15] Centers for Disease Control and Prevention. National Center for Injury Prevention and Control, Division of Violence Prevention. National Violent Death Reporting System Web Coding Manual Version 6.0. Revised January 18, 2022. Accessed February 1, 2024. https://www.cdc.gov/nvdrs/resources/nvdrscodingmanual.pdf

[16] Goldstein EV, Mooney SJ, Takagi-Stewart J, . Characterizing female firearm suicide circumstances: a natural language processing and machine learning approach. Am J Prev Med. 2023;65(2):278-285. doi:10.1016/j.amepre.2023.01.030 36931986

[17] von Elm E, Altman DG, Egger M, Pocock SJ, Gøtzsche PC, Vandenbroucke JP; STROBE Initiative. Strengthening the Reporting of Observational Studies in Epidemiology (STROBE) statement: guidelines for reporting observational studies. BMJ. 2007;335(7624):806-808. doi:10.1136/bmj.39335.541782.AD 17947786 PMC2034723

[18] American Psychiatric Association. Diagnostic and Statistical Manual of Mental Disorders. 5th ed. American Psychiatric Association; 2013.

[19] Arias E, Heron M, Hakes J; National Center for Health Statistics; US Census Bureau. The validity of race and Hispanic-origin reporting on death certificates in the United States: an update. Vital Health Stat 2. 2016;(172):1-21. 28436642

[20] Prater LC, Ellyson AM, Shawon RA, . Suicide, firearms, and terminal illness: a latent class analysis using data from the national violent death reporting system. Psychiatr Serv. 2023;74(6):589-595. doi:10.1176/appi.ps.202100733 36475825

[21] Grant RW, McCloskey J, Hatfield M, . Use of latent class analysis and k-means clustering to identify complex patient profiles. JAMA Netw Open. 2020;3(12):e2029068. doi:10.1001/jamanetworkopen.2020.29068 33306116 PMC7733156

[22] Petersen KJ, Qualter P, Humphrey N. The application of latent class analysis for investigating population child mental health: a systematic review. Front Psychol. 2019;10:1214. doi:10.3389/fpsyg.2019.01214 31191405 PMC6548989

[23] Andersen R, Hagenaars JAP, McCutcheon AL. Applied latent class analysis. Canadian Journal of Sociology/Cahiers Canadiens De Sociologie. 2003;28:584. doi:10.2307/3341848

[24] *Stata Statistical Software*. Version 17.0. StataCorp LLC; 2021.

[25] Kaplan MS, McFarland BH, Huguet N. Characteristics of adult male and female firearm suicide decedents: findings from the National Violent Death Reporting System. Inj Prev. 2009;15(5):322-327. doi:10.1136/ip.2008.021162 19805601

[26] Mann JJ, Apter A, Bertolote J, . Suicide prevention strategies: a systematic review. JAMA. 2005;294(16):2064-2074. doi:10.1001/jama.294.16.2064 16249421

[27] Anglemyer A, Horvath T, Rutherford G. The accessibility of firearms and risk for suicide and homicide victimization among household members: a systematic review and meta-analysis. Ann Intern Med. 2014;160(2):101-110. doi:10.7326/M13-1301 24592495

[28] Betz ME, Miller M, Barber C, . Lethal means access and assessment among suicidal emergency department patients. Depress Anxiety. 2016;33(6):502-511. doi:10.1002/da.22486 26989850 PMC4800489

[29] Miller M, Hemenway D. The relationship between firearms and suicide: a review of the literature. Aggress Violent Behav. 1999;4(1):59-75. doi:10.1016/S1359-1789(97)00057-8

[30] Romero MP, Wintemute GJ. The epidemiology of firearm suicide in the United States. J Urban Health. 2002;79(1):39-48. doi:10.1093/jurban/79.1.39 11937614 PMC3456384

[31] Xiao Y, Bi K, Yip PSF, . Decoding suicide decedent profiles and signs of suicidal intent using latent class analysis. JAMA Psychiatry. 2024;81(6):595-605. doi:10.1001/jamapsychiatry.2024.0171 38506817 PMC10955339

[32] Boggs JM, Beck A, Ritzwoller DP, Battaglia C, Anderson HD, Lindrooth RC. A quasi-experimental analysis of lethal means assessment and risk for subsequent suicide attempts and deaths. J Gen Intern Med. 2020;35(6):1709-1714. doi:10.1007/s11606-020-05641-4 32040838 PMC7280370

[33] Anestis MD, Bryan CJ, Capron DW, Bryan AO. Lethal means counseling, distribution of cable locks, and safe firearm storage practices among the Mississippi national guard: a factorial randomized controlled trial, 2018-2020. Am J Public Health. 2021;111(2):309-317. doi:10.2105/AJPH.2020.306019 33351652 PMC7811068

[34] Walley AY, Paasche-Orlow M, Lee EC, . Acute care hospital utilization among medical inpatients discharged with a substance use disorder diagnosis. J Addict Med. 2012;6(1):50-56. doi:10.1097/ADM.0b013e318231de51 21979821 PMC6034987

[35] Liu L, Zhang C, Nahata MC. Trends in treatment need and receipt for substance use disorders in the US. JAMA Netw Open. 2025;8(1):e2453317. doi:10.1001/jamanetworkopen.2024.53317 39761049 PMC11704973

[36] Hogan MF, Grumet JG. Suicide prevention: an emerging priority for health care. Health Aff (Millwood). 2016;35(6):1084-1090. doi:10.1377/hlthaff.2015.1672 27269026

[37] Mann JJ, Michel CA, Auerbach RP. Improving suicide prevention through evidence-based strategies: a systematic review. Focus (Am Psychiatr Publ). 2023;21(2):182-196. doi:10.1176/appi.focus.23021004 37201140 PMC10172556

[38] Marshall CA, Crowley P, Carmichael D, . Effectiveness of suicide safety planning interventions: a systematic review informing occupational therapy. Can J Occup Ther. 2023;90(2):208-236. doi:10.1177/00084174221132097 36324257 PMC10189833

[39] McGrath RL, Shephard S, Hemmings L, Verdon S, Parnell T. Preventing suicide: time to mobilize the physical therapist workforce. Phys Ther. 2023;103(11):pzad116. doi:10.1093/ptj/pzad116 37622921

[40] Prater LC, Simonetti JA, Knoepke CE, . Older adults and planning for firearm safety: a qualitative study of healthcare providers. J Am Geriatr Soc. 2023;71(4):1275-1282. doi:10.1111/jgs.18188 36550590 PMC10089957

[41] Ross R, Prater LC, Cole A, . Provider perspectives on addressing firearm safety with older adults in primary care. Clin Gerontol. 2024;47(4):555-570. doi:10.1080/07317115.2023.2264291 37791738 PMC10991080

[42] Ehtemam H, Sadeghi Esfahlani S, Sanaei A, . Role of machine learning algorithms in suicide risk prediction: a systematic review-meta analysis of clinical studies. BMC Med Inform Decis Mak. 2024;24(1):138. doi:10.1186/s12911-024-02524-0 38802823 PMC11129374

[43] Nock MK, Millner AJ, Ross EL, . Prediction of suicide attempts using clinician assessment, patient self-report, and electronic health records. JAMA Netw Open. 2022;5(1):e2144373. doi:10.1001/jamanetworkopen.2021.44373 35084483 PMC8796020

[44] Mezuk B, Kalesnikava VA, Kim J, Ko TM, Collins C. Not discussed: inequalities in narrative text data for suicide deaths in the National Violent Death Reporting System. PLoS One. 2021;16(7):e0254417. doi:10.1371/journal.pone.0254417 34270588 PMC8284808

