# Supplementary material for: Mental and Physical Health-Related Risk Factors Among Females Who Died by Firearm Suicide
Source: JAMA Netw Open. 2025 Apr 18;8(4):e255941. doi: 10.1001/jamanetworkopen.2025.5941 (PMC12008759; doi:10.1001/jamanetworkopen.2025.5941)
Supplement: Supplement 1. — eTable. States and Years Reporting Into Study Period, 2014-2018 [file jamanetwopen-e255941-s001.pdf]

Supplemental Online Content

Prater LC, Noghrehchi P, Duan N, et al. Mental and physical health-related risk factors among females who died from firearm suicide. *JAMA Netw Open*. 2025;8(4):e255941. doi:10.1001/jamanetworkopen.2025.5941

eTable. States and Years Reporting Into Study Period, 2014-2018

This supplemental material has been provided by the authors to give readers additional information about their work.

© 2025 Prater LC et al. *JAMA Network Open*.

eTable. States and years reporting into study period, 2014-2018

State	Years
Alabama	2018
Alaska	All
Arizona	2015-2018
California	2017-2018
Colorado	All
Connecticut	2015-2018
Delaware	2017-2018
Georgia	All
Hawaii	2015-2016
Illinois	2016-2018
Indiana	2016-2018
Iowa	2016-2018
Kansas	2015-2018
Kentucky	All
Louisiana	2018
Maine	2015-2018
Maryland	All
Massachusetts	All
Michigan	All
Minnesota	2015-2018
Missouri	2018
Nebraska	2018
Nevada	2017-2018
New Hampshire	2015-2018
New Jersey	All
New Mexico	All
New York	2015-2018
North Carolina	All
Ohio	All
Oklahoma	All
Oregon	All
Pennsylvania	2016-2018
Puerto Rico	2017
Rhode Island	All
South Carolina	All
Utah	All
Vermont	2015-2018
Virginia	All
Washington	2016-2018
West Virginia	2017-2018
Wisconsin	All

© 2025 Prater LC et al. *JAMA Network Open*.
