# Supplementary material for: Mental and Physical Health-Related Risk Factors Among Females Who Died by Firearm Suicide
Source: JAMA Netw Open. 2025 Apr 18;8(4):e255941. doi: 10.1001/jamanetworkopen.2025.5941 (PMC12008759; doi:10.1001/jamanetworkopen.2025.5941)
Supplement: Supplement 2. — Data Sharing Statement [file jamanetwopen-e255941-s002.pdf]

Data Sharing Statement

Prater. Mental and Physical Health-Related Risk Factors Among Females Who Died by Firearm Suicide. *JAMA Netw Open*. Published April 18, 2025.
doi:10.1001/jamanetworkopen.2025.5941

Data
Data available: No

Additional Information
Explanation for why data not available: Data are available through a restricted access agreement with the Center's for Disease Control and Prevention. Data Sharing Agreements indicate we are unable to share raw data. We are happy to provide code as an appendix, if desired.
